# A Rapid Molecular Test for Determining *Yersinia pestis* Susceptibility to Ciprofloxacin by the Quantification of Differentially Expressed Marker Genes

**DOI:** 10.3389/fmicb.2016.00763

**Published:** 2016-05-19

**Authors:** Ida Steinberger-Levy, Ohad Shifman, Anat Zvi, Naomi Ariel, Adi Beth-Din, Ofir Israeli, David Gur, Moshe Aftalion, Sharon Maoz, Raphael Ber

**Affiliations:** Department of Biochemistry and Molecular Genetics, Israel Institute for Biological ResearchNess-Ziona, Israel

**Keywords:** antibiotics, rapid AST, *Y. pestis*, qRT-PCR, molecular testing, ciprofloxacin, minimal inhibitory concentration (MIC), RNA expression

## Abstract

Standard antimicrobial susceptibility tests used to determine bacterial susceptibility to antibiotics are growth dependent and time consuming. The long incubation time required for standard tests may render susceptibility results irrelevant, particularly for patients infected with lethal bacteria that are slow growing on agar but progress rapidly *in vivo*, such as *Yersinia pestis*. Here, we present an alternative approach for the rapid determination of antimicrobial susceptibility, based on the quantification of the changes in the expression levels of specific marker genes following exposure to growth-inhibiting concentrations of the antibiotic, using *Y. pestis* and ciprofloxacin as a model. The marker genes were identified by transcriptomic DNA microarray analysis of the virulent *Y. pestis* Kimberley53 strain after exposure to specific concentrations of ciprofloxacin for various time periods. We identified several marker genes that were induced following exposure to growth-inhibitory concentrations of ciprofloxacin, and we confirmed the marker expression profiles at additional ciprofloxacin concentrations using quantitative RT-PCR. Eleven candidate marker transcripts were identified, of which four mRNA markers were selected for a rapid quantitative RT-PCR susceptibility test that correctly determined the Minimal Inhibitory Concentration (MIC) values and the categories of susceptibility of several *Y. pestis* strains and isolates harboring various ciprofloxacin MIC values. The novel molecular susceptibility test requires just 2 h of antibiotic exposure in a 7-h overall test time, in contrast to the 24 h of antibiotic exposure required for a standard microdilution test.

## Introduction

Rapid determination of the antibiotic susceptibility of pathogenic bacteria is required to assure proper treatment of the bacterial infection. Bacterial susceptibility is determined by standard antimicrobial susceptibility tests (ASTs) performed according to Clinical and Laboratory Standards Institute guidelines (CLSI, 2015). ASTs are growth-dependent assays in which bacterial growth in the presence and absence of the relevant antibiotic is compared. Preliminary bacterial isolation, enrichment and identification steps are required. Thus, susceptibility determination is a time-consuming procedure that may delay appropriate antibiotic treatment for the patient, and a novel rapid AST is needed. Rapid ASTs are particularly important for fatal pathogenic bacteria that grow slowly *in vitro* but rapidly *in vivo*, such as *Yersinia pestis*.

*Y. pestis*, a Gram-negative bacterium, is the etiological agent of plague, a severe and rapidly progressing disease characterized by a high mortality rate (40–60% for the bubonic, 30–50% for the septicemic, and 100% for the pneumonic forms of plague) if not treated with proper antibiotics within 18–24 h after the onset of symptoms (Pollitzer, 1960; Inglesby et al., 2000). Plague has caused millions of deaths in three world pandemics, and local outbreaks in endemic areas (Gage and Kosoy, 2005; Butler, 2013) or potential intentional dissemination in a bioterror event remain concerns. Due to its rapid disease progression and person-to-person transmission (Inglesby et al., 2000), *Y. pestis* is classified by the Centers for Disease Control and Prevention (CDC) as a Tier 1 Select Agent. To prevent morbidity and mortality, rapid identification of the microbial agent and its susceptibility to recommended antibiotics is needed. The recommended antibiotics for post-exposure prophylaxis of *Y. pestis* are ciprofloxacin, doxycycline and, as an alternative, chloramphenicol, whereas the recommended antibiotics for treatment are streptomycin, gentamicin and, as alternatives, levofloxacin, ciprofloxacin, doxycycline, moxifloxacin and chloramphenicol (Inglesby et al., 2000; CDC, 2016^1^). Most naturally occurring *Y. pestis* strains are susceptible to the recommended antibiotics; however, plasmid-mediated single and multiple drug-resistant strains have been isolated from infected patients (Galimand et al., 2006). To select appropriate antibiotics for plague prophylaxis or treatment, a microdilution test is recommended (CLSI, 2015). *Y. pestis* growth in liquid is not significantly slower than other Gram-negative bacterial pathogens; however, its growth on solid agar, which is required for isolation, is considered slow. Therefore, the standard procedure for a positive clinical sample usually requires 3 days (including 2 days for the enrichment step on rich agar followed by 24 h for the AST). Moreover, ASTs of environmental *Y. pestis* samples (as in a bioterrorism attack) require an additional isolation step of the sample on selective agar and thus require at least 5 days (including 2 days for the isolation step, 2 days for the enrichment step, and 24 h for the AST). Because death may occur within a few hours of bacterial exposure and symptom onset (Pollitzer, 1960; Dennis and Hughes, 1997), rapid ASTs are urgently needed.

In recent years, different novel methods have been developed to decrease the AST duration by reducing the time required for either the preliminary isolation and enrichment steps or the susceptibility determination step (reviewed by Pulido et al., 2013; van Belkum and Dunne, 2013). For example, we have developed a rapid susceptibility test for *Y. pestis*-positive blood cultures that includes the direct isolation and enrichment of the bacteria from the blood culture components using a vacutainer serum separation tube, followed by bacterial counting using flow cytometry (Steinberger-Levy et al., 2007). Few of the novel alternative methods [such as flow cytometry for live/dead determination (Jepras et al., 1997)] enable the determination of both the antibiotic minimal inhibitory concentration (MIC) and category of susceptibility, and most other methods [such as using MALDI-TOF-MS (Burckhardt and Zimmermann, 2011) or magnetic bead rotation (Kinnunen et al., 2012)] determine only the category of susceptibility (reviewed by van Belkum and Dunne, 2013). Determining the MIC value of antibiotics and not only the susceptibility category is particularly important in complicated cases, such as infected patients with contraindications to the first-choice antibiotic, pregnant women, children and strains whose sensitivity is close to the resistant category break-point (intermediate category).

In the present study, we describe the development of a rapid mRNA-based molecular AST using *Y. pestis* and ciprofloxacin as our bacteria:antibiotic model combination. The molecular AST is based on monitoring the changes in the expression of specific mRNA transcripts (mRNA markers) induced by 2-h exposure to ciprofloxacin.

Ciprofloxacin, the model antibiotic in the present study, is a bactericidal fluoroquinolone. Fluoroquinolones inhibit bacterial growth mainly by binding to DNA gyrase and topoisomerase IV and thus preventing DNA synthesis and repair, resulting in DNA damage and bacterial death (Redgrave et al., 2014). Exposure of various bacteria to ciprofloxacin or other fluoroquinolones induces common transcriptomic changes, such as induction of genes belonging to the SOS response pathway, as well as changes in mRNA transcripts that differ among studies (Gmuender et al., 2001; Shaw et al., 2003; Kaldalu et al., 2004; Brazas and Hancock, 2005; Cirz et al., 2006, 2007). Moreover, the fold induction of different genes varies among studies. These differences can be attributed to the different experimental conditions used, such as bacterial species, antibiotic concentrations, antibiotic exposure time, inoculum size and growth medium. In addition, previous transcriptomic analyses were not performed under standard CLSI-recommended conditions.

Thus, to identify marker mRNA transcripts that are altered upon *Y. pestis* exposure to inhibitory concentrations (1 × MIC and above) of ciprofloxacin, we conducted transcriptome analysis using DNA microarray analysis. The exposure experiments were performed under CLSI-recommended conditions. Several mRNA transcripts that were induced or repressed, depending on both exposure time and ciprofloxacin concentration, were identified. Using 4 marker genes, we developed a 7-h quantitative RT-PCR (qRT-PCR)-based AST for the determination of *Y. pestis* susceptibility to ciprofloxacin. The measured changes in transcription levels were translated to the strain's MIC value and the susceptibility category. The molecular-derived MIC values were similar to those obtained using the standard 24-h CLSI test. Our rapid molecular AST was confirmed using the virulent Kimberley53 strain, its non-virulent derivative Kimberley53pCD1^−^pPCP1^−^, several Kimberley53pCD1^−^pPCP1^−^ isolates with reduced susceptibility toward ciprofloxacin and the EV76 vaccine strain.

## Materials and methods

### Bacterial strains and growth conditions

Experiments were conducted using the virulent *Y. pestis* strain Kimberley53 (Ben-Gurion and Hertman, 1958). An in-house DNA sequencing analysis of this strain (which harbors the 3 virulent plasmids: pMT1, pCD1, and pPCP1) revealed 99.7% homology with the CO92 clinical strain (biovar orientalis) and 100% identity in the sequences encoding known virulent factors, including both chromosomal and plasmidial factors. Kimberley53 has been shown to be virulent in both pneumonic (Vagima et al., 2015) and bubonic (Tidhar et al., 2009) mouse plague models, and its mouse LD_50_ in those models resembles the CO92 MLD_50_ values. In addition, we also used the plasmid-cured non-virulent Kimberley53pCD1^−^pPCP1^−^ derivative strain (Flashner et al., 2004) or the vaccine strain EV76 (Ben-Gurion and Hertman, 1958). Experiments using the virulent strain were performed using biosafety level 3 (BSL-3) containment and procedures. Experiments conducted using the non-virulent strains, were performed using biosafety level 2 (BSL-2) containment and procedures.

Kimberley53pCD1^−^pPCP1^−^ spontaneous mutants with reduced susceptibility to ciprofloxacin were isolated according to previously published reports (Lindler et al., 2001; Udani and Levy, 2006; Louie et al., 2007a,b, 2011a,b). The isolation was performed in compliance with Israeli law for working with select agents, approved by the Institutional “Recombinant DNA Experimental Usage Committee” and performed according to specific Institutional “Biosafety committee” guidelines for containment and working procedures.

Bacteria were plated on brain-heart infusion agar (BHIA, BD) or on BIN [a *Y. pestis* selective agar plate (Ber et al., 2003)]. Live counting of bacteria was performed by plating serial 10-fold dilutions on BHIA, followed by incubation for 48 h. All incubations were conducted at the optimal growth temperature of 28°C.

### Standard ciprofloxacin MIC determination

ASTs were performed by standard microdilution test (CLSI, 2015) in a 96-well microplate (TPP) using an inoculum containing 5 × 10^5^–1 × 10^6^ colony-forming units (CFUs)/ml suspended in cation-adjusted Mueller-Hinton broth (MHB, BBL). Ciprofloxacin (Ciproxin 200, Bayer) was serially diluted two-fold in MHB to a final concentration in the range of 0.001–16 μg/ml. Bacterial cultures were incubated for 24 h at 28°C in a plate reader (Sunrise or Infinite 200, TECAN), and the optical density at 630 nm (OD_630_) was read at 1-h intervals. The MIC value was defined after 24 h of growth as the lowest ciprofloxacin concentration that reduced growth to less than 10% of the OD_630_ of the no-antibiotic growth control. No growth was verified by unaided visual inspection. Each assay was performed with three independent experiments. The microdilution kinetic curves represent the average absorbance of three well replicates for each antibiotic concentration. The error bars represent the standard deviation (SD).

### Antibiotic exposure

*Y. pestis* exposed to ciprofloxacin was prepared for either transcriptomic DNA microarray analysis or molecular-based AST by suspending BHIA- or BIN-plated (lawn with pinhead-sized colonies) bacteria in MHB to OD_660_ = 0.1 (1–2 × 10^8^ CFU/ml). The cultures were then diluted 1:200 in MHB to obtain the standard CLSI-recommended inoculum of 5 × 10^5^–1 × 10^6^ CFU/ml (CLSI, 2015).

For the transcriptomic DNA microarray analysis, BHIA-plated virulent Kimberley53 was suspended in MHB (in five 2-l Erlenmeyer flasks each containing 420 ml of standard inoculum). The flasks were incubated with continuous shaking at 150 rpm for 2 h for the adjustment of the culture to the standard AST MHB medium. The cultures were then combined, mixed and divided into five culture samples of 400 ml each. Ciprofloxacin stock solutions (4 ml of 100 ×) were added to four cultures to obtain final concentrations of 0.001, 0.016, 0.5, and 4 μg/ml, and the non-treated culture was used as a growth control. Each of the five cultures was then further divided into 4 × 100 ml in 0.5-l flasks (each flask represents one exposure time point). Samples (0.5 ml) were taken for microdilution AST (triplicates of 0.1 ml/well) to confirm the standard MIC value (0.15 ml of the 0.016 μg/ml sample was also diluted 1:1 to verify the partial inhibition of growth at the sub-MIC concentration of 0.008 μg/ml; see **Figure 2A**). The 0.5-l Erlenmeyer flasks were incubated with shaking at 150 rpm for either 20, 45, 90, or 120 min. At each time point, samples were taken for both live counting and total RNA extraction. For RNA extraction, 40 ml of the bacterial suspension was centrifuged at 3200 × g for 15 min at 4°C, the supernatant was discarded, and the bacterial pellet was frozen by liquid nitrogen and stored at −70°C until RNA extraction.

To prepare the inoculum for the molecular AST experiments, bacteria were plated on BIN for 20–24 h. The inoculum was then prepared and recovered for 2 h in MHB prior to ciprofloxacin exposure as described above. The recovered cultures were exposed to serial 2-fold dilutions of ciprofloxacin (in the range of 0.002–16 μg/ml, as indicated in the figures) in MHB for 2 h in a sample volume of 0.5 ml in a 24-well flat bottom plate (Costar 3524). The entire 0.5-ml well volume was used for RNA extraction.

### RNA purification

Total RNA was extracted from bacterial pellets using the RNeasy Mini kit (QIAGEN) according to the manufacturer's instructions. Residual DNA was removed by 15-min on-column digestion using the RNase-free DNase kit (QIAGEN). RNA samples were quantified using a NanoDrop ND-1000 spectrophotometer. For DNA microarray analysis, the quality of the RNA samples was determined using an Agilent 2100 Bioanalyzer with the “Prokaryote Total RNA Nano” chip. The RNA integrity number (RIN value) was determined for each sample, and samples with RIN ≥ 9 were used for the DNA microarray analysis. RNA samples were stored at −70°C until further use.

### Transcriptomic microarray analysis

Complementary RNA (cRNA) was produced from the RNA samples and fluorescently labeled using the MessageAmp™ II-Bacteria kit (Ambion) according to the manufacturer's instructions with either Cy3-CTP or Cy5-CTP (Perkin Elmer) as the fluorescent label. Specific activity and cRNA concentrations were determined using a NanoDrop ND-1000 spectrophotometer. The labeled samples were stored at −70°C until use. Dual-color DNA microarray hybridizations were performed using a custom Agilent 8x15K slide containing probes for the 4196 chromosomal and plasmidial *Y. pestis* CO92 genes and pseudogenes [Accession no. NC_003143.1 NCBI; (Parkhill et al., 2001)]. Hybridization and scanning were performed as suggested by Agilent. The slides were scanned in the Agilent DNA microarray scanner G2505B. Images were analyzed, and data were extracted using the Agilent Feature Extraction (FE) software (version 9.5.1.1), with linear and lowess normalization. Statistical analysis was performed using the Limma (Linear Models for Microarray Data) package from the Bioconductor project (http://www.bioconductor.org). The processed signal from the FE was read into Limma using the “read.maimages” function. Background subtraction and lowess normalization were performed for each array. Quantile normalization was applied between arrays. The Benjamini-Hochberg false discovery rate (FDR) was used to correct for multiple comparisons. The fold change (FC) in each gene was calculated as the median FC value measured for 3–5 different or replicated probes representing the same gene. Each experiment comprised 4 samples representing 4 different ciprofloxacin concentrations and labeled with Cy3-CTP that were co-hybridized with the growth control sample, which was not exposed to ciprofloxacin and was labeled with Cy5-CTP. Each slide was hybridized with samples originating from two independent biological experiments representing the same time point, performed with Cy3-Cy5 dye swap. The results are available online (http://www.ncbi.nlm.nih.gov/geo/), GEO ID: GSM2101145.

### Quantitative reverse transcription-PCR (qRT-PCR)

One-step qRT-PCR was performed in a 50-μl reaction mixture containing 1 ng of total RNA as the template, 1 × buffer (0.5 M KCl, 0.1 M Tris-HCl, pH 8.8 [Bio-Rad], 1 μM SuperROX® [Biosearch Technologies]), 0.6 μM each primer, 0.3 μM TaqMan probe, 0.2 mM dNTP, 3 mM MgCl_2_, 0.5 μl of Sensiscript reverse transcriptase (QIAGEN), 2 U of Taq DNA polymerase (Promega) and 0.4 μl of JumpStart Taq antibodies (Sigma). Gene-specific primers and TaqMan probes (5′ 6-FAM; 3′ BHQ-1) were designed based on the *Y. pestis* CO92 genomic sequence (NC_003143.1) using Primer Express software (Table S1) and were ordered from IDT (USA). qRT-PCR was performed in an Applied Biosystems 7500 real-time PCR system under the following conditions: 50°C for 30 min, 95°C for 3 min, and 40 cycles of 94°C for 15 s and 60°C for 35 s. Negative template controls (NTCs) were used for each primer/probe set to exclude nonspecific reactions. No RT controls lacking Sensiscript reverse transcriptase were used to confirm the effective removal of genomic DNA. A standard curve was obtained for each primer/probe set using a serial 10-fold dilution of the growth control RNA sample (100–0.01 ng/reaction) to confirm the high dynamic range of their reaction efficiency (results not shown).

The transcript FC, defined as the ratios of the mRNA expression level in the ciprofloxacin-exposed sample to that in the untreated sample, was calculated from the difference between the Ct values (as obtained from the 7500 Real-time PCR System Sequence Detection Software) of the ciprofloxacin-exposed samples (Ct _treatment_) and the Ct value of the untreated control sample (Ct _control_) using the equation: FC = 2^−ΔCt^ (ΔCt = Ct _treatment_−Ct _control_).

For RNA extracted from 0.5-ml culture volumes (where RNA concentrations were below the NanoDrop ND-1000 limit of detection), equal volumes (5 μl) of total RNA were used for the qRT-PCR, and the RNA concentrations were normalized using a 16S rRNA primer set (Table S1) with a 1:1000 dilution of the samples. FC was calculated according to the ΔΔCt method (FC = 2^−ΔΔCt^ where ΔΔCt = ΔCt _marker gene_−ΔCt_16S RNA_).

Representative outcomes from two independent experiments are presented in the figures.

## Results

### Identification of ciprofloxacin growth inhibitory-responsive genes by transcriptomic screening

**Figure 1:**
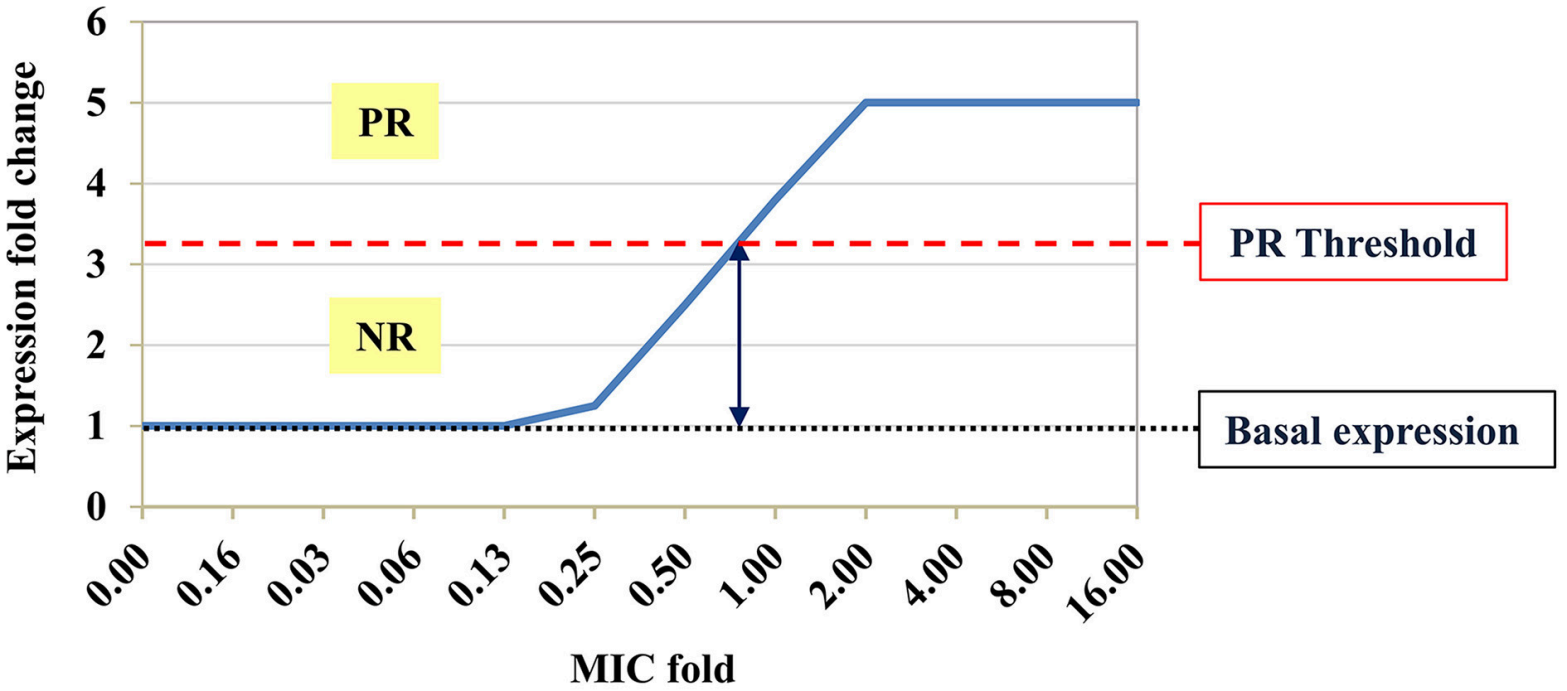
**Schematic expression profile of a potential marker gene**. Hypothetical pattern of a potential marker gene expression profile following exposure to an antibiotic at sub-MIC, MIC and above the MIC. Basal expression of the tested marker gene in the growth control sample (black dotted line) was defined as fold change (FC) = 1. Expression FC < threshold value (red dashed line) was defined as a negative response (NR), whereas FC ≥ threshold value was defined as a positive response (PR) to the antibiotic. The MIC derived from the molecular AST is the minimal antibiotic concentration that leads to PR.

**Figure 2:**
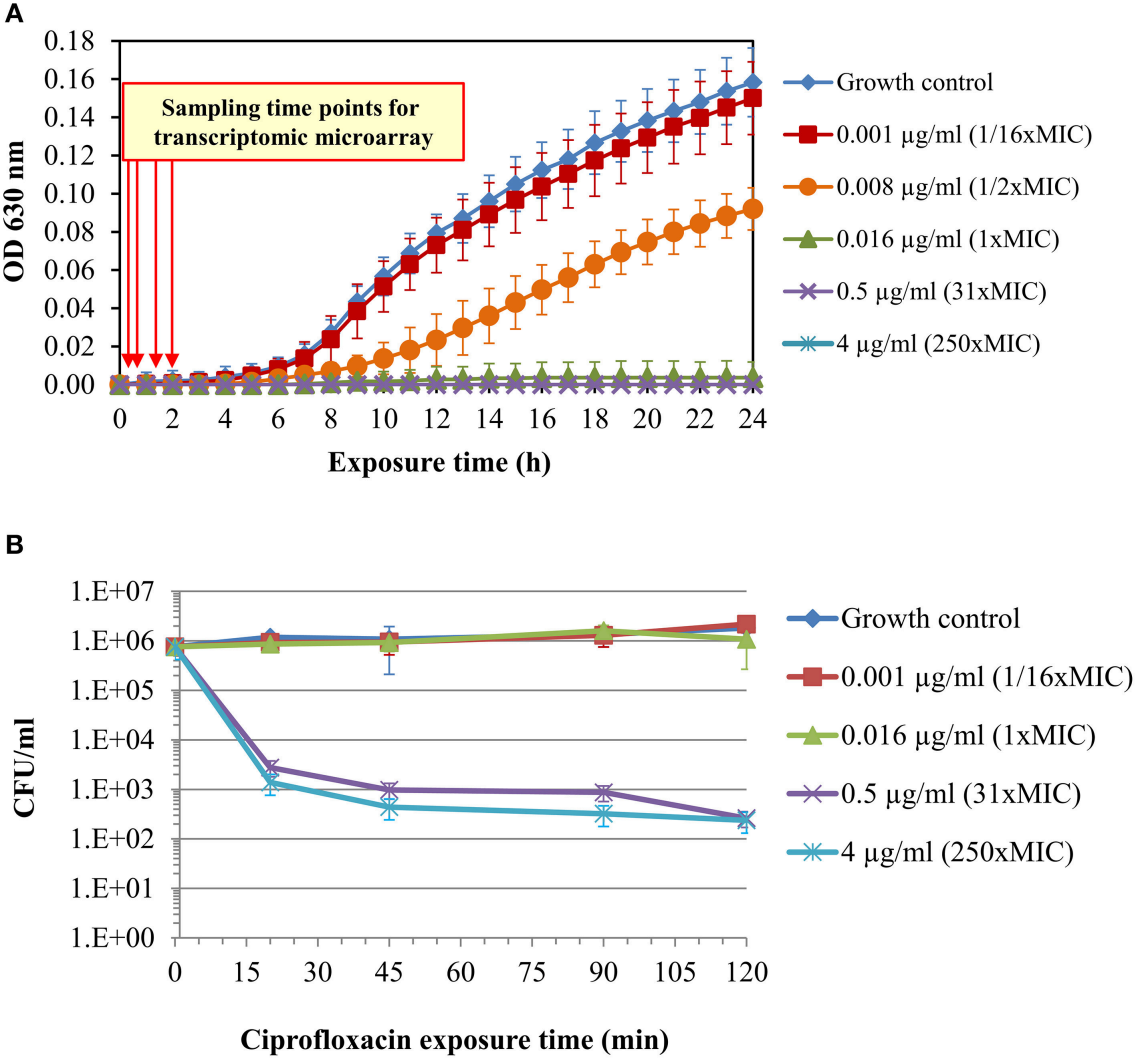
**Standard microdilution test and survival curves of ***Y. pestis*** Kimberley53 exposed to ciprofloxacin**. A culture of MHB-suspended *Y. pestis* Kimberley53 was prepared from BHIA-plated bacteria to a standard inoculum size of 5 × 10^5^–1 × 10^6^ CFU/ml and incubated for 2 h at 28°C with moderate agitation (for adjustment of the culture from the BHIA to MHB growth medium) before performing the 24-h microdilution test. **(A)** The microdilution test was conducted in a 96-well microplate at the indicated ciprofloxacin concentrations, and the OD_630_ was measured hourly using a TECAN plate reader. **(B)** Survival analysis was performed by BHIA plating of serial 10-fold dilutions of replicate samples at different time points after ciprofloxacin addition. The error bars represent the SD of 3 independent experiments.

To develop a rapid qRT-PCR-based molecular AST, we searched for candidate marker genes whose expression is altered in correlation with the susceptibility level following short exposure of *Y. pestis* to growth-inhibitory concentrations (≥1 × MIC) of ciprofloxacin (see schematic presentation in Figure 1). For this purpose, an inoculum of 5 × 10^5^–1 × 10^6^ CFU/ml of *Y. pestis* Kimberley53 virulent strain was obtained from BHIA, suspended in MHB and incubated for 2 h at 28°C before exposure to different ciprofloxacin concentrations, for different incubation times. Incubations were performed at the optimal growth temperature of 28°C. Because inoculum size influences the MIC value (as illustrated for *Y. pestis* exposed to ciprofloxacin in Figure S1), we used an inoculum concentration of 5 × 10^5^–1 × 10^6^ CFU/ml as recommended by the CLSI guidelines (CLSI, 2015). Cultures were exposed to ciprofloxacin concentrations of 0.001, 0.016, 0.5, and 4 μg/ml (equivalent to 1/16 ×, 1 ×, 31 ×, and 250 × MIC, respectively). Samples of the exposed cultures were withdrawn after 20, 45, 90, and 120 min. Total RNA purification and the transcriptomic profile characterization were performed as described in the Materials and Methods. The standard MIC value was determined in parallel using the microdilution susceptibility test and the same inoculum, culture media, and antibiotic solutions (Figure 2A). In addition, live bacterial counting was performed to characterize the ciprofloxacin-induced bacterial growth arrest and cell death and its correlation with the transcriptomic alterations following 2-h antibiotic exposure (Figure 2B).

Live CFU counting of a culture exposed to a sub-MIC concentration of ciprofloxacin (0.001 μg/ml; 1/16 × MIC) did not reveal growth inhibition or cell death (Figure 2). In contrast, exposure to 1 × MIC (0.016 μg/ml) ciprofloxacin led to growth arrest (Figure 2A), while exposure to the higher ciprofloxacin concentrations of 0.5 and 4 μg/ml (31 × and 250 × MIC, respectively) caused both growth arrest (Figure 2A) and a rapid ~3 log reduction of bacterial CFU counts (Figure 2B). The survival curves of the 31 × and 250 × MIC-exposed bacteria exhibited typical biphasic patterns, consistent with the rapid killing of most cells by the fluoroquinolone and a persistent subpopulation that can survive the bactericidal effect of the antibiotic (Dörr et al., 2009).

The transcriptomic analysis of the 1 × MIC ciprofloxacin-exposed culture revealed changes in the expression (both up-regulation and down-regulation by a FC ≥ 2) of 0, 3, 13, and 31 genes following 20, 45, 90, and 120 min of ciprofloxacin exposure, respectively (Table 1). However, only some of the genes (3, 7, and 13 for 45, 90, and 120 min of ciprofloxacin exposure, respectively) exhibited changes in expression levels following exposure to 1 × MIC as well as to 31 × and 250 × MIC. Because more candidate marker genes and higher expression FC values were observed following 2 h of exposure to 1 × MIC ciprofloxacin compared to the other time points, we further developed the rapid molecular susceptibility assay using a 2-h incubation period. After 2 h of exposure, 11 genes were induced and 2 genes were repressed by a FC > 2 in all of the three tested ciprofloxacin concentrations. One of the 11 induced genes, YPMT1.86A, is plasmid encoded and was excluded from further analysis because the plasmid may not be present in all *Y. pestis* strains. Notably, 8 of the 10 chromosomal induced genes belong to the SOS response gene family (*recN, dinI* [YPO1232], *recA, oraA, dinI* [YPO1586], b4058, *dinP*, and b4043), which is known to be induced following DNA damage caused by ciprofloxacin. Similar effects have been reported following exposure of heterologous bacteria to various fluoroquinolones (Gmuender et al., 2001; Shaw et al., 2003; Kaldalu et al., 2004; Cirz et al., 2006, 2007).

**Table 1:** **Transcriptomic microarray results for the 1 × MIC responsive genes^**a**^**.

	**Gene Id^b^**	**Gene name^b^**	**Annotation^b^**	**Median fold change**^**d**^								
				**45 min**.			**90 min**.			**120 min**.		
				**MIC fold**			**MIC fold**			**MIC fold**		
				**1x**	**31x**	**250x**	**1x**	**31x**	**250x**	**1x**	**31x**	**250x**
Induced genes	YPO1105	*recN*	DNA repair protein RecN	2.2	5.3	17.3	3.7	4.3	18.5	4.1	5.2	12.0
	YPO3307	*lexB*	RecA	2	6	15.4	2.9	4.6	12.7	3.1	4.8	11.7
	YPO1436	*sfiA*	Putative cell division inhibitor	2	2.7	2.1	2.7			3.5		2.2
	YPO1231	*pla2*	Putative outer membrane-associated protease				3.1	5.3	61	4.6	10.6	58.7
	YPO3306	*oraA*	Putative regulatory protein				2.4	4.6	24.7	2.7	10.5	33.9
	YPO1232	*dinI*	Putative stress response protein				2.3	7.4	41	3.7	9.0	49.8
	YPO0324	b4058	Excinuclease ABC subunit A				2	2.2	5.6	2.3	3.0	5.0
	YPPCP1.05^c^	*pst*	Pesticin				3.1		2.2	5.1		
	YPO0518	*dinA*	DNA polymerase II				2.4		2.9	2.6		2.5
	YPO3231	*dinP*	DNA-damage-inducible protein P				2.3		3	2.6	2.3	3.3
	YPO3841	*mutU*	DNA helicase II				2.2			2.5		
	YPO1586	*dinI*	DNA-damage-inducible protein I							2.9	3.3	13.0
	YPO1233	YPO1233	Putative prophage repressor protein							2.6	4.8	10.6
	YPMT1.86A^c^	YPMT1.86A	Hypothetical protein							2.5	4.1	7.1
	YPO0314	b4043	LexA repressor							2.7	3.4	3.7
	YPO3782	*rumC*	Putative DNA recombination protein							2.3		
	YPO3901	*ilvG*	Acetolactate synthase isozyme II large subunit							2.2		
	YPO4111	YPO4111	Putative periplasmic solute-binding protein							2.2		
	YPO3900	*ilvM*	Acetolactate synthase isozyme II small subunit							2.2		
	YPO3899	*ilvE*	Branched-chain amino acid aminotransferase							2.1		
	YPO0003	*asnA*	Aspartate-ammonia ligase							2.1	2.1	
	YPO3914	*sth*	Soluble pyridine nucleotide transhydrogenase							2.1		
	YPO4119	*glmU*	UDP-N-acetylglucosamine pyrophosphorylase							2.0		
	YPO4110	YPO4110	ABC transporter permease							2.0		
	YPO3015	*cysP*	Thiosulfate-binding protein							2.0		
	YPO3872	*rep*	ATP-dependent DNA helicase Rep							2.0		
Repressed genes	YPO2140	YPO2140	Hypothetical phage protein				−2.2					
	YPO1398	*cspB*	Cold shock-like protein				−2.7	−8.2	−12	−2.5	−7.4	−10.4
	YPO2659	*cspB*	Cold shock protein							−2.1	−5.5	−10.7
	YPO2711	*rpoE*	RNA polymerase sigma E factor							−2.0		−4.2
	YPO2712	*mclA*	Sigma E factor negative regulatory protein							−2.0		−5.2
	YPO1325	YPO1325	Putative membrane protein							−2.2		

a *The table lists genes that exhibited at least a 2-fold difference in expression and an adjusted p-value (FDR) < 0.05. Genes that were changed following exposure to 31 × MIC and or 250 × MIC but not following 1 × MIC ciprofloxacin are not listed in the table*.

b *Gene ID, gene name and annotation are based on Y. pestis CO92 data (NC 003141.1, Parkhill et al., 2001)*.

c *Plasmidial gene (all other genes are chromosomal)*.

d The median fold change color is scaled as follows:

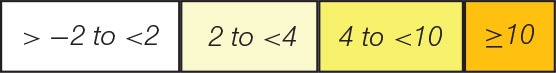

The DNA microarray screen results were validated by qRT-PCR analysis of the 10 induced and the two repressed genes using the same RNA samples that were used for the DNA microarray analysis. For comparison, a gene identified as non-responsive in the DNA microarray analysis (*capR* [YPO3155]) was included in the qRT-PCR validation. Seven of the 10 induced chromosomal genes (*pla2, recN, dinI* [YPO1232], *recA, oraA, dinI* [YPO1586] and YPO1233) exhibited significant changes in expression using both techniques (Figure S2 and Table 2). A good correlation with the DNA microarray results was also observed for the two repressed genes. The non-responsive gene, *capR*, displayed some reduction in expression at the higher ciprofloxacin concentrations in the qRT-PCR analysis, which may be attributed to the different normalization processes of the two quantification methods.

**Table 2:** **Comparison of the FCs of selected genes determined by transcriptomic microarray and qRT-PCR analysis**.

	**MIC fold**	DNA microarray results^*^			qRT-PCR results^**^		
		**1x**	**31x**	**250x**	**1x**	**31x**	**250x**
Induced genes	*pla2* [YPO1231]	4.6	10.6	58.7	4.4	9.1	157.4
	*recN* [YPO1105]	4.1	5.2	12.0	3.3	2	4.7
	*dinI* [YPO1232]	3.7	9.0	49.8	3.2	4.4	20.9
	*recA* [YPO3307]	3.1	4.8	11.7	4.2	4.8	6.6
	*oraA* [YPO3306]	2.7	10.5	34.0	2.6	2.9	16.2
	*dinI* [YPO1586]	2.9	3.3	13.0	3.1	1.5	12.1
	YPO1233	2.6	4.8	10.6	2.1	1.7	4.9
	b4058 [YPO0324]	2.3	3.0	5.0	1.5	1.0	1.9
	b4043 [YPO0314]	2.7	3.4	3.7	2.1	1.5	1.2
	*dinP* [YPO3231]	2.6	2.3	3.3	1.8	0.8	1.0
Repressed genes	*cspB-like* [YPO1398]	−2.5	−7.4	−10.4	−2.8	−15	−23.8
	*cspB* [YPO2659]	−2.1	−5.5	−10.7	−1.9	−7	−21.5
Non-responsive gene	*capR* [YPO3155]	1.0	1.0	1.0	−1.2	−2.9	−3.3

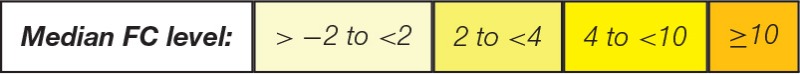

* The DNA microarray values are derived from the 2-h exposure data in Table 1. The values represent the calculated median FC as described in the Materials and Methods.

** *The qRT-PCR values represent the mean values as described in the Materials and Methods*.

For use in a molecular AST, we chose to focus on positive response monitoring of chromosomal genes; thus, the 7 induced potential marker genes were selected for further characterization in a microdilution test format.

### Characterization of the expression profile of the candidate marker genes in a microdilution format

Microdilution is the standard 24-h incubation test recommended by the CLSI for susceptibility determination of *Y. pestis* (CLSI, 2015). To shorten this AST, we developed a molecular AST based on the standard microdilution format, which requires only a 2 h ciprofloxacin-exposure period, followed by RNA purification and qRT-PCR monitoring of the FC levels of the marker genes. The assay was performed in adherence to CLSI-recommended conditions to maximize the possibility of obtaining MIC values that are similar (equal or within one 2-fold dilution) to the standard microdilution-derived MIC values. The molecular AST-derived MIC was defined as the minimal antibiotic concentration that causes changes in the marker gene expression levels equal to or above a threshold level (see schematic presentation in Figure 1). The threshold values used in the molecular ASTs were determined by analyzing results at 1 × MIC from all of the exposure experiments using different hypothetical threshold values (starting with a value of 2.0 with 0.1 increments). For each marker, we chose the threshold value that maximized the correct MIC prediction for all tested strains while giving preference to minimizing major errors that lead to a lower molecular MIC prediction as compared to the actual MIC result by standard microdilution. We hypothesized that these threshold values could be used to determine the MICs for *Y. pestis* strains with unknown susceptibility levels.

**Figure 3:**
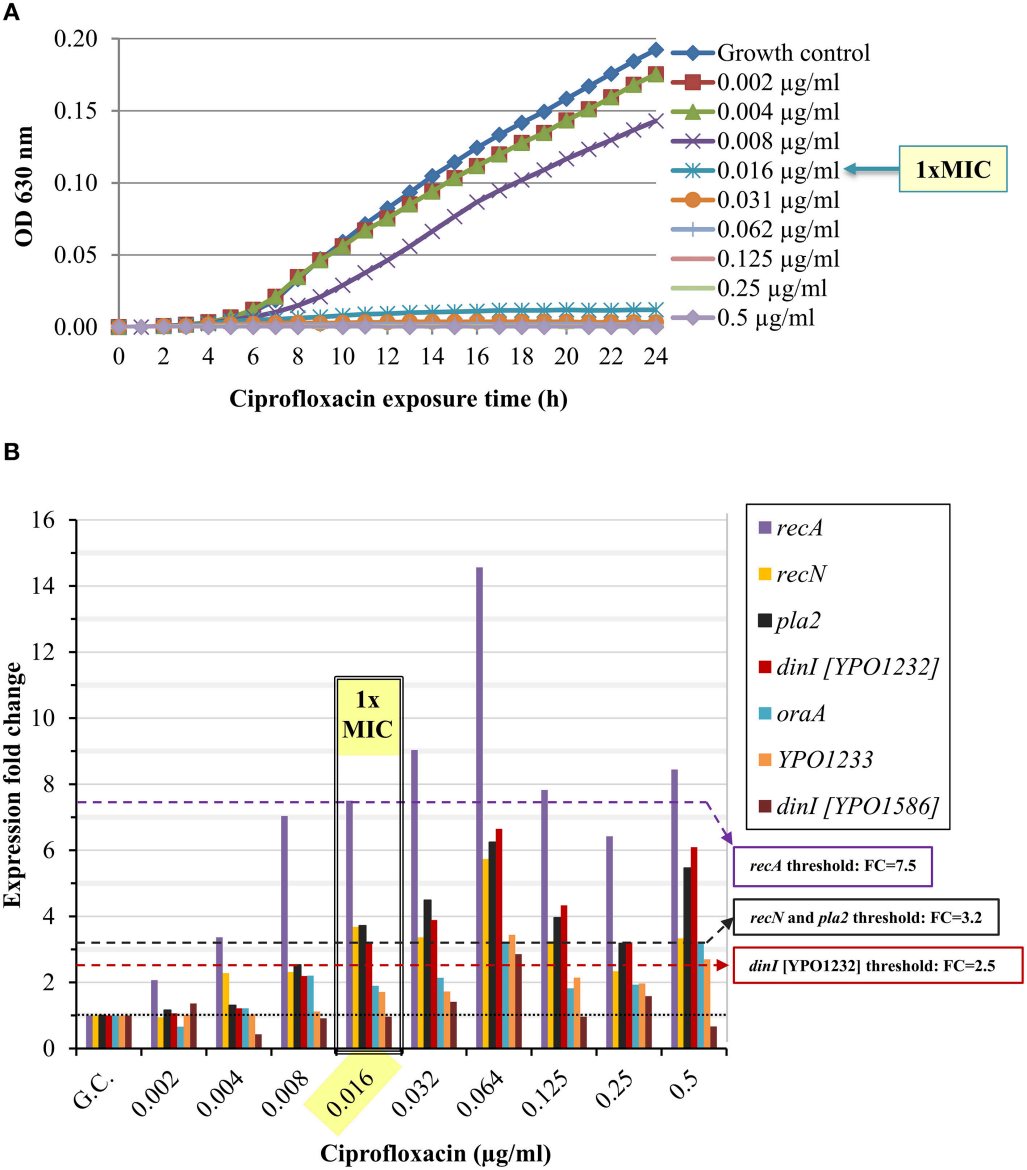
**Ciprofloxacin-induced growth inhibition and changes in the expression levels of the candidate genes**. BHIA-isolated colonies of *Y. pestis* Kimberley53 were suspended in MHB to a standard inoculum size of 5 × 10^5^ to 1 × 10^6^ CFU/ml. The culture was incubated for 2 h of recovery at 28°C followed by exposure to 2-fold serial dilutions of ciprofloxacin (40 ml each) at a range of *0.002–0.5* μg/ml. **(A)** Samples of 100 μl were transferred (from the 40 ml ciprofloxacin-exposed cultures) to a 96-well microplate. The optical density at 630 nm was recorded hourly for 24 h using a TECAN plate reader. The MIC value was determined at the 24-h time point. The OD_630_ values are the average of 3 replicated wells. **(B)** Cultures (40 ml) exposed to different concentrations of ciprofloxacin for 2 h, were used for total RNA extraction, and 1 ng of RNA sample was analyzed by qRT-PCR. The black dotted line represents basal expression (*FC* = 1) in the growth control sample, and the colored dashed lines represent the threshold values of the tested marker genes. The figure is representative of duplicate analyses.

We determined the standard MIC and the expression profile of the candidate marker genes following *Y. pestis* Kimberley53 exposure to serial two-fold dilutions of ciprofloxacin, yielding a range of antibiotic concentrations around the MIC (0.002–0.5 μg/ml, representing 0.125 × –31 × MIC). The MIC value of ciprofloxacin in the standard 24-h microdilution test for Kimberley53 was 0.016 μg/ml (Figure 3A). Exposure to sub-MIC concentrations of ciprofloxacin (0.002–0.008 μg/ml, 0.125–0.5 × MIC, respectively) resulted in a slight reduction of the growth rate compared to the growth control (Figure 3A). Monitoring the changes in the candidate marker gene expression levels revealed that 4 of the 7 candidate marker genes (*recA, recN, pla2*, and *dinI* [YPO1232]) were induced > 2-fold following exposure to ciprofloxacin concentrations of 0.016 μg/ml (1 × MIC) and above. However, the bacteria also responded to the sub-MIC concentrations with variable but correlated levels of induction. These results emphasize the high sensitivity of the molecular test, which monitors the direct and primary effects of the antibiotic on the bacterial molecular response, whereas the growth rate indicates the consequence of those molecular alterations. For example, *recA* was induced 2-fold following exposure to a sub-MIC concentration of 0.002 μg/ml (0.125 × MIC, Figure 3B). The induction was enhanced as the ciprofloxacin concentration increased. A threshold value of 7.5 was assigned for *recA* usage as a marker gene for 1 × MIC value identification. Another candidate gene, *recN*, was induced ~2-fold following exposure to a sub-MIC concentration of 0.004 μg/ml (0.25 × MIC), and a threshold FC value of 3.2 was set for the 1 × MIC value determination (Figure 3B). Similarly, we determined the threshold values for *pla2*, which was induced ~2-fold upon exposure to 0.008 μg/ml (0.5 × MIC) ciprofloxacin, and a threshold of 3.2 was assigned for the determination of the 1 × MIC value (Figure 3B). A threshold FC of 2.5 was assigned to *dinI* [YPO1232]. Thus, four candidate genes, *recA, recN, pla2* and *dinI* [YPO1232], exhibited potential utility as marker genes, while the compiled expression profile results disqualified the other potential markers.

### Confirmation of the molecular AST using Kimberley53pCD1^−^pPCP1^−^ derivatives with decreased ciprofloxacin susceptibility

For the proof-of-concept of the qRT-PCR-based molecular AST, we analyzed the gene expression profiles of various non-virulent *Y. pestis* Kimberley53pCD1^−^pPCP1^−^ derivatives with different ciprofloxacin MIC values. The derivatives were either ciprofloxacin sensitive (MIC ≤ 0.25 μg/ml) or non-sensitive (MIC > 0.25 μg/ml), as defined by the ciprofloxacin susceptibility category for *Y. pestis* (CLSI, 2015). Point mutations in the quinolone resistance-determining region (QRDR) locus, which is part of ciprofloxacin target encoding, the *gyr*A gene, are known to be acquired following exposure to fluoroquinolones and reduce the bacterial sensitivity to the antibiotic (Wiedemann and Heisig, 1994; Redgrave et al., 2014). Therefore, we sequenced the QRDR locus of these isolates. Several point mutations were identified (Table 3), thus partially explaining the reduced sensitivity of these derivatives to ciprofloxacin.

**Table 3:** **Characterization of the reduced ciprofloxacin sensitivity of non-virulent Kimberley53pCD1^**−**^pPCP1^**−**^ derivatives**.

**Derivative #**	**MIC value (μg/ml)^a^**	**CLSI Category of susceptibility**	***gyrA* mutation**
Kimberley53pCD1^−^pPCP1^−^	0.016	Sensitive	w.t.
#83	0.125	Sensitive	D87G
#34	0.25	Sensitive	D82N
#111	0.5	Non-S	S83R
#66	1	Non-S	S83I
#66-6	4	Non-S	S83I

*Non-S, non-sensitive*.

a *MIC values were determined using the standard microdilution susceptibility test*.

**Figure 4:**
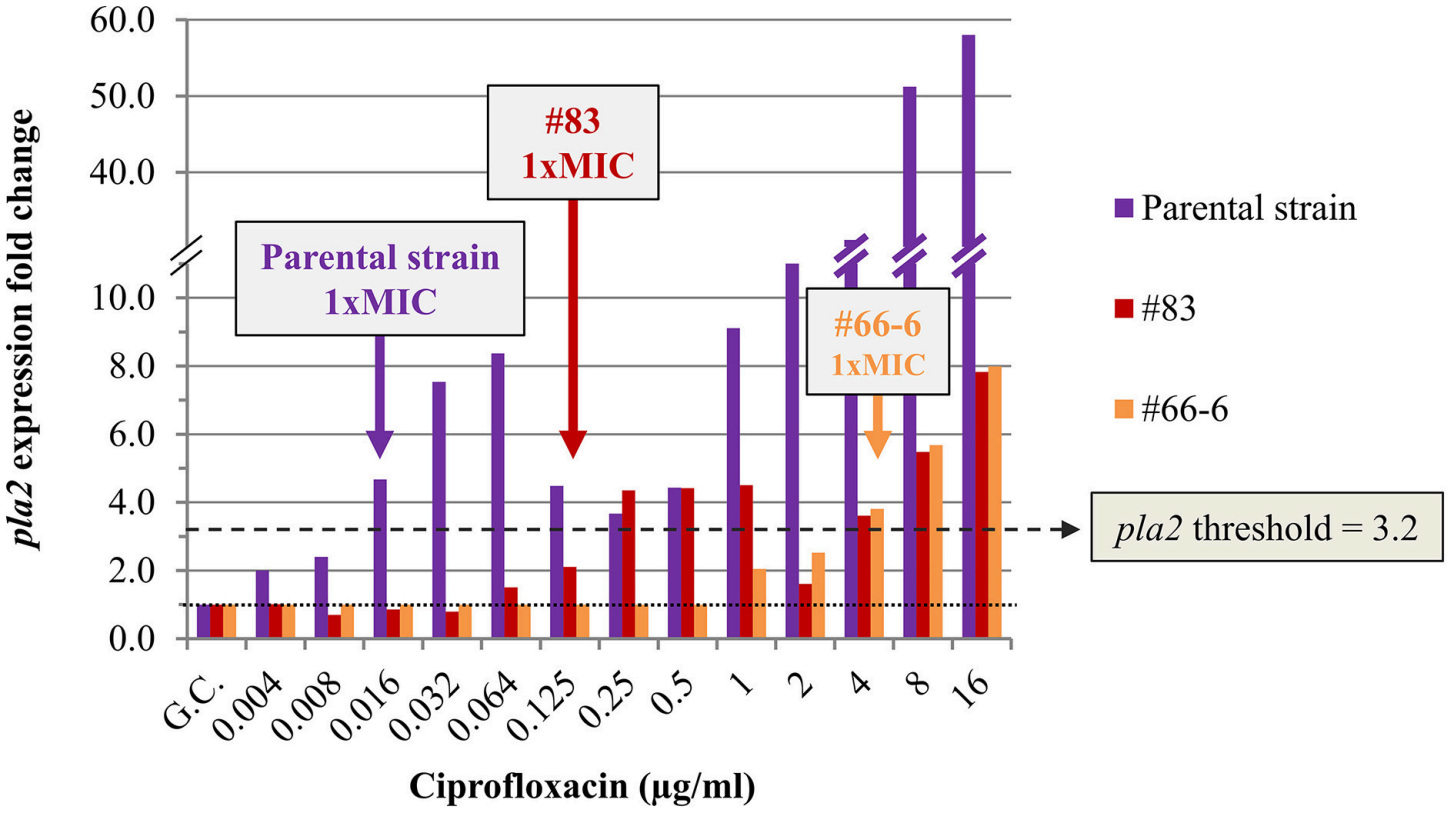
**Expression of ***pla2*** in non-virulent ***Y. pestis*** Kimberley53pCD1^**−**^pPCP1^**−**^ and its derivatives #83 and #66-6**. The expression profile of *pla2* was determined in the non-virulent *Y. pestis* Kimberley53pCD1^−^pPCP1^−^ (parental strain) and its ciprofloxacin reduced-susceptibility derivatives #83 and #66-6. Cultures were prepared from bacteria grown overnight on BIN, suspended in MHB and incubated for 2 h followed by 2 h exposure to the required ciprofloxacin concentrations in a 0.5-ml culture volume in 24-well plates. RNA samples were purified and used for qRT-PCR analysis using 16S rRNA as a reference for FC determination as described in the Materials and Methods. The MIC value for each derivative was also determined by a standard microdilution test performed using the same cultures that were used for the molecular AST. The colored arrows indicate the microdilution-measured MIC values of the bacterial derivatives. The black dotted line represents basal expression (*FC* = 1) in the growth control sample, and the green dashed line represents the threshold value assigned to *pla2* (*FC* = 3.2). The figure is representative of duplicate analyses.

**Figure 5:**
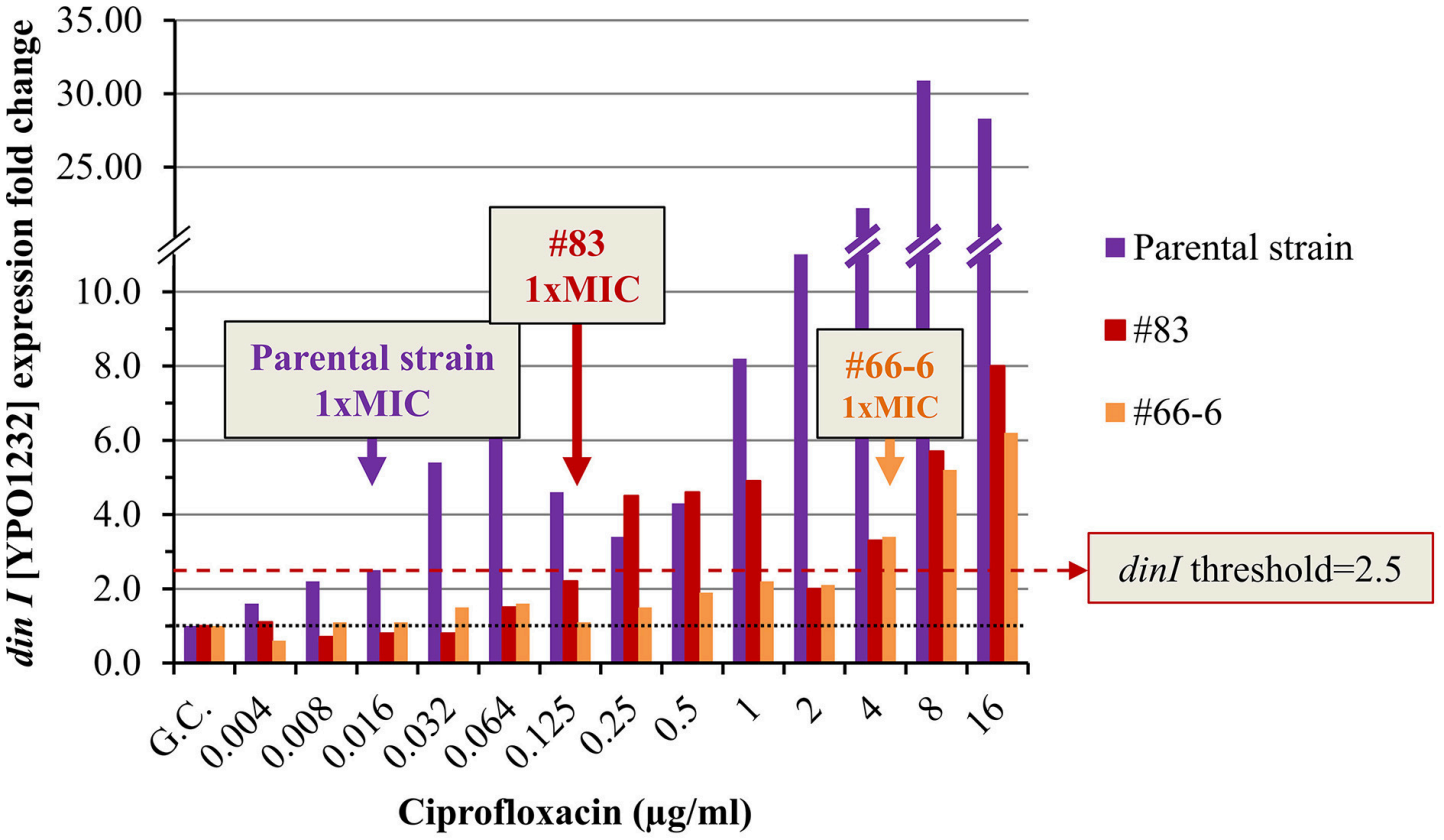
**Expression pattern of ***dinI [YPO1232]*** in non-virulent ***Y. pestis*** Kimberley53pCD1^**−**^pPCP1^**−**^ and its derivatives #83 and #66-6**. The molecular AST was performed as described in Figure 4. The black dotted line represents basal expression (*FC* = 1) in the growth control sample, and the red dashed line represents the threshold value assigned to *dinI* [YPO1232] (*FC* = 2.5). The figure is representative of duplicate analyses.

The expression profiles of the four potential marker genes (*recA, recN, pla2*, and *dinI* [YPO1232]) in the non-virulent Kimberley53pCD1^−^pPCP1^−^ parental strain exposed to ciprofloxacin, resembled those in the w.t. virulent Kimberley53 strain (compare Figure S3 with Figure 3B). The *pla2* gene was induced ~4.5-fold in the parental strain exposed to 0.016 μg/ml ciprofloxacin (microdilution measured 1 × MIC value), representing an FC above the assigned threshold value (*FC* = 3.2), as required (Figure 4 and Figure S3). In contrast, isolates #83 and #66-6, which have higher MIC values (0.125 and 4 μg/ml, respectively), exhibited no induction at that (0.016 μg/ml) concentration (Figure 4). However, exposure to higher ciprofloxacin concentrations representing the isolates' higher MIC values caused induction near the threshold value and above. The exposure of isolate #83–0.125 μg/ml ciprofloxacin (the MIC value of this isolate), resulted in a lower level of induction compared to the parental strain (*FC* = 2.1 vs. 4.7). Thus, using a threshold value of 3.2 for *pla2*, determined previously, led to a molecular MIC of 0.25 μg/ml compared to 0.125 μg/ml observed in the microdilution test. A two-fold difference is acceptable because in the standard microdilution test, the accepted MIC value for QC reference strains might be in the range of 1 to 2 two-fold dilutions (CLSI, 2015). The non-sensitive isolate #66-6 (standard microdilution MIC = 4 μg/ml) exhibited marker induction above the threshold only following exposure to ciprofloxacin at its MIC value (Figure 4). Similar results were obtained in the qRT-PCR-based AST using *dinI* [YPO1232] (Figure 5).

**Figure 6:**
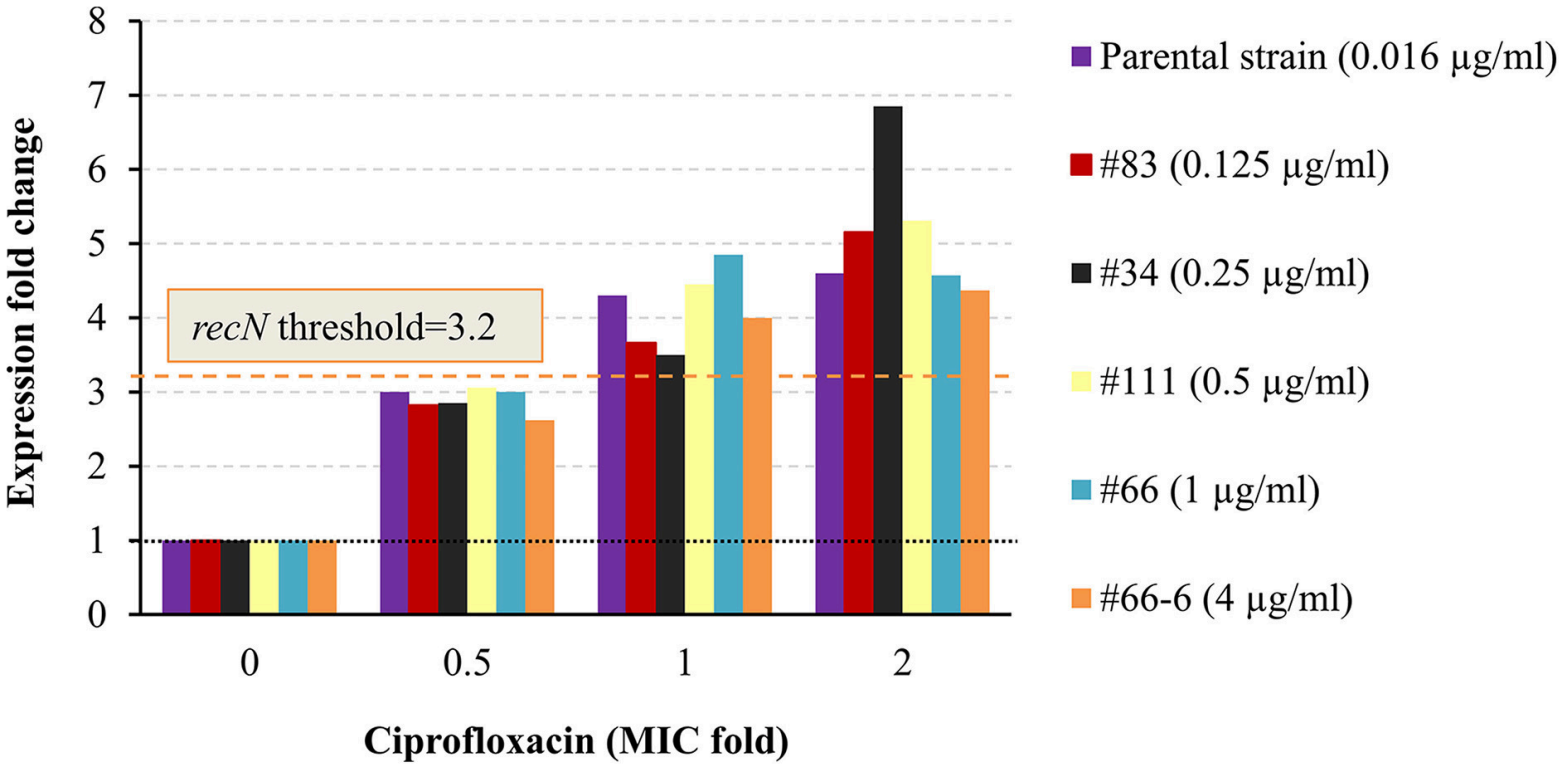
**Expression pattern of ***recN*** in non-virulent ***Y. pestis*** EV76 and Kimberley53pCD1pPCP1^**−**^ and its derivatives**. The molecular AST was performed as described in Figure 4. For each derivative, the corresponding MIC (as determined by the standard microdilution assay) is given in parenthesis. The black dotted line represents basal expression (*FC* = 1) in the growth control sample, and the orange dashed line represents the threshold value assigned to *recN* (*FC* = 3.2). The figure is representative of duplicate analyses.

The level of induction of an additional marker gene, *recN*, in the different isolates exposed to 0.5 ×, 1 ×, and 2 × MIC ciprofloxacin was correlated with the isolate's MIC value. The threshold of 3.2 enabled the determination of the 1 × MIC values of both the sensitive and the non-sensitive *Y. pestis* isolates (Figure 6).

The ability of the qRT-PCR assay to determine the MIC value was also tested with EV76, another *Y. pestis* strain. The expression pattern of the four potential marker genes (*recA, recN, pla2*, and *dinI* [YPO1232]) was similar to the pattern observed for Kimberley53 and its derivative (Figure S4 vs. Figure 3B and Figure S3). Using the above-mentioned assigned thresholds for *recN* and *dinI* [YPO1232]) enabled the determination of the correct MIC value (microdilution MIC = 0.031 μg/ml). The MIC value determined using the threshold assigned for *pla2* was 0.064 μg/ml (2 × microdilution MIC), which is within an acceptable range of the AST results. It should be noted that even though the expression pattern of *recA* was similar to that of the other marker genes, the previously assigned threshold for *recA* (7.5) could not be used to define the MIC value for EV76 because its expression level in this strain was lower than that in Kimberley53 and its derivative Kimberley53pCD1^−^pPCP1^−^. The threshold value assigned for *recA* was high due to its high expression level, i.e., in sub-MIC concentrations of 0.5 × and 0.25 × MIC, in Kimberley53 and its derivative Kimberley53pCD1^−^pPCP1^−^ (Figure 3B and Figure S3), indicating that not only the threshold level but also the pattern of the markers' response should be taken into consideration when predicting bacterial susceptibility. In such cases, we could use the other 3 marker genes (*recN, pla2*, and *dinI* [YPO1232]) for MIC determination and the expression pattern of the fourth marker gene (*recA*) to strengthen the MIC interpretation.

In this manuscript, we present a proof-of-concept for rapid determination of antibiotic MIC value using a molecular approach. However, validation of the assay should be done by using additional *Y. pestis* strains, with different genetic backgrounds and biovars. This will enable the recommendation of valid threshold values for the candidate marker genes and the determination of the minimal marker genes required for valid MIC interpretation.

Our results indicate that the qRT-PCR AST based on these mRNA markers has the potential to be used for the rapid determination of the MIC values of ciprofloxacin for *Y. pestis*.

## Discussion

Reducing the time required for ASTs is of significant importance, particularly for patients infected with pathogenic bacteria that grow slowly *in vitro* but can cause rapidly progressing fatal disease, such as that caused by *Y. pestis*. In addition, the emerging number of drug-resistant bacterial strains necessitates the development of novel technologies for rapid ASTs (Pulido et al., 2013; van Belkum and Dunne, 2013). Standard ASTs are time consuming because they are based on bacterial growth in the presence and absence of the tested antimicrobial agent in liquid media or growth in antibiotic diffusion gradients, such as the disk diffusion test or Etest methods (Jorgensen and Ferraro, 2009). In addition to the time required for incubation during the antibiogram tests, a pure culture must first be obtained by isolation and enrichment steps. These growth-dependent and time-consuming steps are the bottleneck for standard ASTs and prolong the overall process to several days and even weeks, depending on the bacterial source and the growth rate of the tested bacteria. Thus, the development of alternative rapid ASTs is crucial.

Various alternative AST methods have been described in recent years, including nucleic acid-based ASTs. These nucleic acid-based ASTs are performed by determining either the bacterial chromosomal DNA copy number using qPCR or phage-specific mRNA transcript copy number using qRT-PCR (Waldeisen et al., 2011; Mulvey et al., 2015). However, these assays are still growth dependent and time consuming. PCR methods to detect mutations or gene lesions responsible for the acquisition of resistance have been used for various bacteria, such as monitoring point mutations in *gyrA* responsible for ciprofloxacin resistance in *Y. pestis* (Lindler and Fan, 2003). However, this method is not reliable because only some resistance-conferring genes are known. Moreover, the MIC value cannot be determined because the correlation of the bacterial genotype with the antibiotic susceptibility is not direct (van Belkum and Dunne, 2013).

In the present article, we describe the development of a rapid molecular AST that is based on quantifying the FC of specific mRNA marker expression following antibiotic exposure. In contrast to DNA-based methods, our approach is correlated with the level of bacterial susceptibility to the tested antibiotic agent. If the bacteria are sensitive to the tested antimicrobial concentration, the culture will exhibit a physiological response according to its susceptibility, and this response can be quantified at the mRNA level. However, if the bacteria are not sensitive to the tested antimicrobial concentration, no stress will be imposed on the culture; thus, no significant change in the expression of the specific marker genes will be observed. Accordingly, at higher antibiotics concentrations equal to or above the MIC of the reduced susceptibility strain, a significant change in the markers' expression will be observed, thus enabling the determination of the bacterial MIC value.

Antibiotic-responsive (induced or repressed) genes were previously described for various antimicrobial agents; however, these genes were not universal and not necessarily suitable for MIC determination. The development of a molecular test requires the identification of marker genes suitable for the specific drug-bacterium combination and under the specific (standard) exposure conditions. Therefore, we performed DNA microarray analysis of the *Y. pestis* Kimberley53 virulent strain after exposure for various time periods to several ciprofloxacin concentrations under standard CLSI test guidelines. Ciprofloxacin was chosen because it is one of the antibiotics of choice for treatment in *Y. pestis* exposure or ongoing plague disease (Inglesby et al., 2000; Peterson et al., 2010). We identified several SOS responsive genes that were induced, and their levels of induction were correlated with ciprofloxacin concentration. Some of those genes were also induced to a greater extent as the exposure time increased (Table 1). Eight genes (*recN, dinI* [YPO1232], *recA, oraA, dinI* [YPO1586], b4058, *dinP*, and b4043, Table 1) of the 11 induced genes identified following 2 h of exposure belong to the SOS response gene family. We characterized four genes (*recA, pla2, recN*, and *dinI* [YPO1232]) of the 11 induced genes for potential use as markers for antimicrobial susceptibility determination by the qRT-PCR method. The expression FC value of each marker mRNA following exposure to 1 × MIC antibiotic was determined relative to the basal expression in the growth control and was used for assignment of the threshold value. The markers' threshold values were used to interpret the MIC values of various *Y. pestis* strains and derivatives. The minimal antibiotic concentration that resulted in marker gene expression equal to or greater than its assigned threshold value was determined as the molecular MIC value. This molecular MIC value was identical to or differed by up to one 2-fold dilution from the standard microdilution-derived MIC value and predicted identical ciprofloxacin susceptibility categories of several derivatives, representing a wide range of ciprofloxacin susceptibilities (Figures 4–6). When evaluating a new susceptibility method, both sensitive and resistant strains should be examined, and an overall 1 × MIC agreement of greater than 90% plus or minus one double dilution of a standard CLSI-derived MIC should be attained (Jorgensen and Ferraro, 2009). Our molecular assay is well within the range of these guidelines.

An mRNA-based AST of various heterologous bacteria and antibiotics was recently described (Barczak et al., 2012; Hou et al., 2015). However, the experiments were performed using a non-standard inoculum (OD_600_ of ~1, which represents ~10^9^ CFU/ml). Inoculum concentration affects the MIC value (Figure S1), and thus molecular susceptibility determination using a non-standard inoculum size might be inaccurate. The reported mRNA-based AST enables the determination of the category of susceptibility only and not the MIC value, in contrast to the method described here. In addition, the reported mRNA-based AST was used to monitor alterations in gene expression in cultures exposed to only one representative antibiotic concentration (Barczak et al., 2012). We believe that molecular AST should be conducted using a wider range of 2-fold antibiotic dilutions in a microdilution-like format that includes the category interpretation breakpoints, as we presented here. This format, together with molecular mRNA marker expression changes that correlate with the bacterial susceptibility level, will enable the rapid determination of the category of susceptibility of *Y. pestis* strains, regardless of their MIC value.

**Figure 7:**
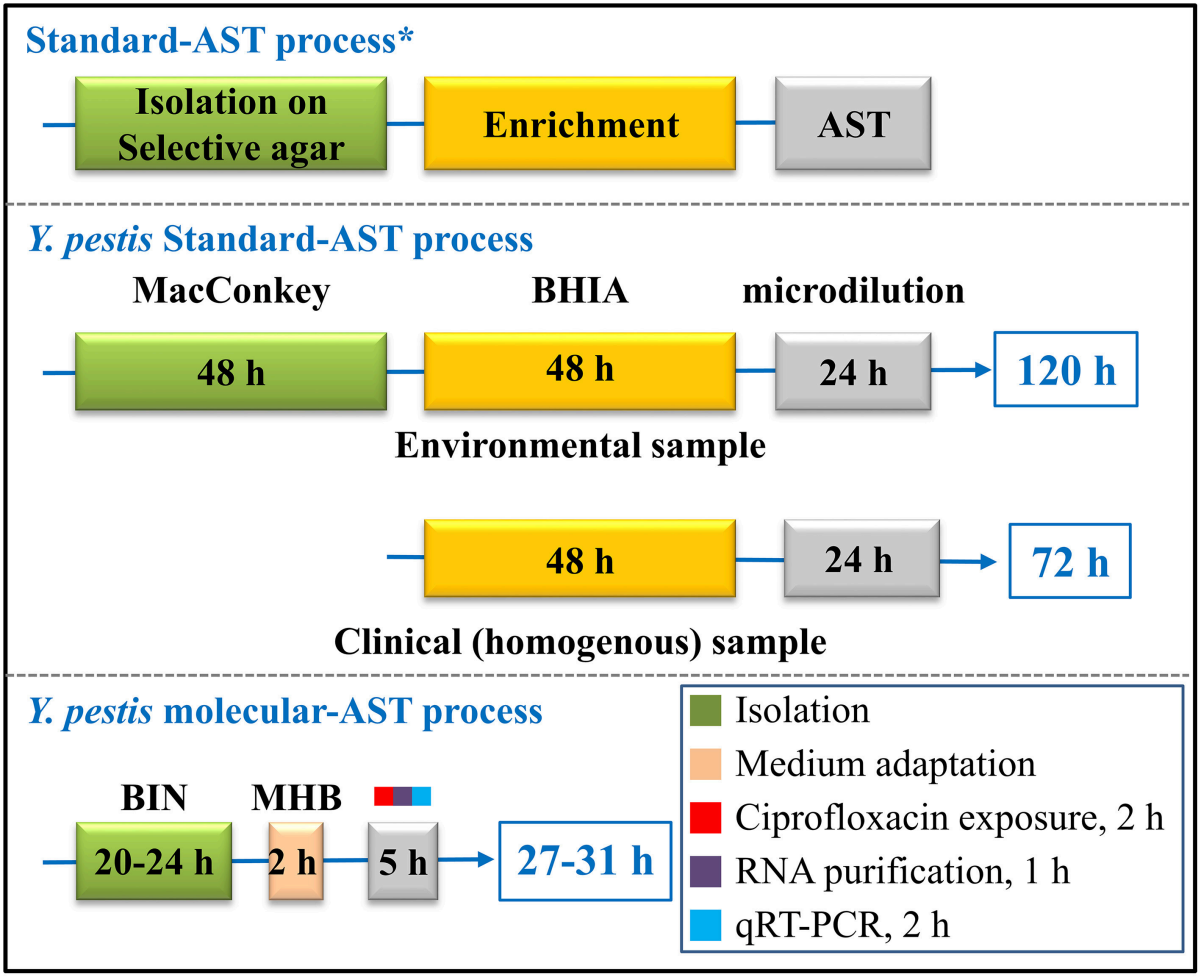
**Schematic comparison of the steps and duration of the standard AST and the molecular AST for ***Y. pestis*** susceptibility determination**. ^*^Isolation and enrichment steps are recommended by the CDC/WHO (Dennis et al., 1999). The AST conditions were guided by the CLSI recommendations (CLSI, 2015).

Here, we demonstrate that the duration of the AST can be reduced by replacing the 24-h standard microdilution test with a 7-h molecular AST that includes the following steps: culture recovery (2 h), ciprofloxacin exposure (2 h), total RNA purification (1 h) and qRT-PCR analysis of specific mRNA markers (2 h; Figure 7). Automation of the total RNA purification and qRT-PCR steps would make the assay simpler and even faster.

Moreover, the duration of the overall susceptibility determination process may be shortened by reducing not only the AST assay but also the isolation and enrichment preliminary steps. This alteration may be achieved using BIN, a selective agar plate that can enable both *Y. pestis* selection and efficient growth (Ber et al., 2003). Here, we used a 20- to 24-h BIN-plated *Y. pestis* as the source for the molecular AST. Thus, the usage of BIN in combination with the molecular AST may reduce the overall required time for the standard CLSI-based AST process, which can be up to 120 h for heterogeneous environmental samples or ~72 h for homogenous clinical samples, to a 27- to 31-h protocol (Figure 7). Moreover, isolation and enrichment from blood samples may be replaced by a 15-min procedure of centrifugation using a vacutainer serum separation tube (vacutainer SST), as we previously reported (Steinberger-Levy et al., 2007) and may thus enable the antibiogram results within 8 h.

A similar approach can be used to develop molecular assays for additional antibiotics and for other slow-growing pathogenic bacteria. We are currently using this novel molecular AST approach to develop a similar assay for MIC and susceptibility category determination of doxycycline for *Y. pestis*, an additional recommended antibiotic for prophylaxis and treatment of plague.

## Author contributions

Research project design: IS, OS, AZ, NA, AB, OI, and RB. Experiments: IS, OS, AZ, NA, DG, MA, SM, and RB. Writing: IS, OS, and RB.

## Funding

This study was supported by the Israeli Institute of Biology Research, Ness Ziona, Israel.

### Conflict of interest statement

The authors declare that the research was conducted in the absence of any commercial or financial relationships that could be construed as a potential conflict of interest.

## Footnotes

^1^http://www.cdc.gov/plague/healthcare/clinicians.html
