# Supplementary material for: A Rapid Molecular Test for Determining Yersinia pestis Susceptibility to Ciprofloxacin by the Quantification of Differentially Expressed Marker Genes
Source: Front Microbiol. 2016 May 19;7:763. doi: 10.3389/fmicb.2016.00763 (PMC4871873; doi:10.3389/fmicb.2016.00763)
Supplement: Supplementary file 1 [file Table1.DOCX]

**Table S1: Primers and probes sequences**

| **Gene name** (amplicon size, bp) | **Gene Id** | **Primer/ TaqMan probe** | **Sequence (5'-->3')** |
| --- | --- | --- | --- |
| ***recN*** (100) | YPO1105 | Forward | TTGGGCCAACATCTAATTCAGA |
|  |  | Reverse | ATTGATTTGCGTAGGCATCCA |
|  |  | probe | CCACGGCCAACACGCTCATCA |
| ***recA*** (97) | YPO3307 | Forward | CTCCCTTTCCCTTGATATTGCAC |
|  |  | Reverse | AATATCTACACCCAATTTCTTGGCA |
|  |  | probe | AGATTGGGTCAAGGGCATGTTCGGC |
| ***pla2***  (100) | YPO1231 | Forward | ACGGATACAGCAGGCAACGT |
|  |  | Reverse | GCTTTTGGTTAGGTTCACTGGATT |
|  |  | probe | CACCTCGCTTGGATTATTGAATGCCG |
| ***dinI*** (102) | YPO1232 | Forward | GCGGAGGTTAACGTCAAAGC |
|  |  | Reverse | ATCAAACATCTCTTCAACCAAACG |
|  |  | probe | TCAATACCAATGCCAGCAAACAAGAAAAGTCA |
| ***oraA*** (100) | YPO3306 | Forward | ACTCTCCCAGCGTGACCATAGT |
|  |  | Reverse | TTTAGCACCTGAACGCTTTCC |
|  |  | probe | CTTGCGGCACCTCCATTTTCGG |
| ***dinI*** (100) | YPO1586 | Forward | TTGCCCGCAGGTGCTATC |
|  |  | Reverse | TCGCATAACGCACCTGTACTG |
|  |  | probe | TAAAAGACTCAATACAAAATTCCCGGACACGG |
| **b4058** (104) | YPO0324 | Forward | GAAGGAACGGTAGATGACATCATG |
|  |  | Reverse | TCAGCACTGACCCGTTTCTCT |
|  |  | probe | CACCGGCGTCACTCACGGGC |
| **b4043** (101) | YPO0314 | Forward | GACCTGGTCCGCGATCAC |
|  |  | Reverse | TGCTCTTCAGCAGCGTTAGG |
|  |  | probe | TAGCGCAAACAGGTATGCCACCGA |
| **YPO1233** (100) | YPO1233 | Forward | ATACGCTGCGTGACATTGAGA |
|  |  | Reverse | GATGGCGAGGTCATGGAATC |
|  |  | probe | TGGCCTGAATCGACCAAGCATGC |
| ***dinP*** (100) | YPO3231 | Forward | GAGGCCTATTTGGATGTTTCTGAT |
|  |  | Reverse | GTCAGGTTCAGCTCACTAGCGATA |
|  |  | probe | CGGTGGCTCTGCAACATTAATCGCTC |
| ***cspB*** (109) | YPO1398 | Forward | TGGTTCGGATGTTTATGTCAACA |
|  |  | Reverse | CGGTCCGTGAATACTACGATATGT |
|  |  | probe | AACGGCCATTGCCAACACCAAAAA |
| ***cspB*** (100) | YPO2659 | Forward | CTGGTTTGGTAAAATGGTTTGATG |
|  |  | Reverse | CCCTGGATTGCAGAGAAATGTAC |
|  |  | probe | AAGGTTTTGGTTTTATTTCTCCTGCAGATGGC |
| ***capR*** (101) | YPO3155 | Forward | GGATTACGATCTCTCGGATGTGA |
|  |  | Reverse | AGCCGGACAGACGAATAACTTC |
|  |  | probe | TTGTGGCGACCTCTAACTCCATGAATATTCC |
| ***16S rRNA*** (155) | Accession Nr_074199.1 | Forward | ACAGAATTTGGCAGAGATGCTAAAG |
|  |  | Reverse | CCTTGAGTTCCCACCATTACGT |
|  |  | probe**^a^** | CACGAGCTGACGACAGCCATGCA |

**^a^**A universal probe (Young *et al.*, 2000).

**SUPPLEMENTAL REFERENCE**

Young, G. M., Badger, J. L., Miller, V. L. (2000). Motility is required to initiate host cell invasion by *Yersinia enterocolitica*. *Infect. Immun.* 68, 4323-4326.
