# Supplementary figures and images for: A Rapid Molecular Test for Determining Yersinia pestis Susceptibility to Ciprofloxacin by the Quantification of Differentially Expressed Marker Genes
Source: Front Microbiol. 2016 May 19;7:763. doi: 10.3389/fmicb.2016.00763 (PMC4871873; doi:10.3389/fmicb.2016.00763)

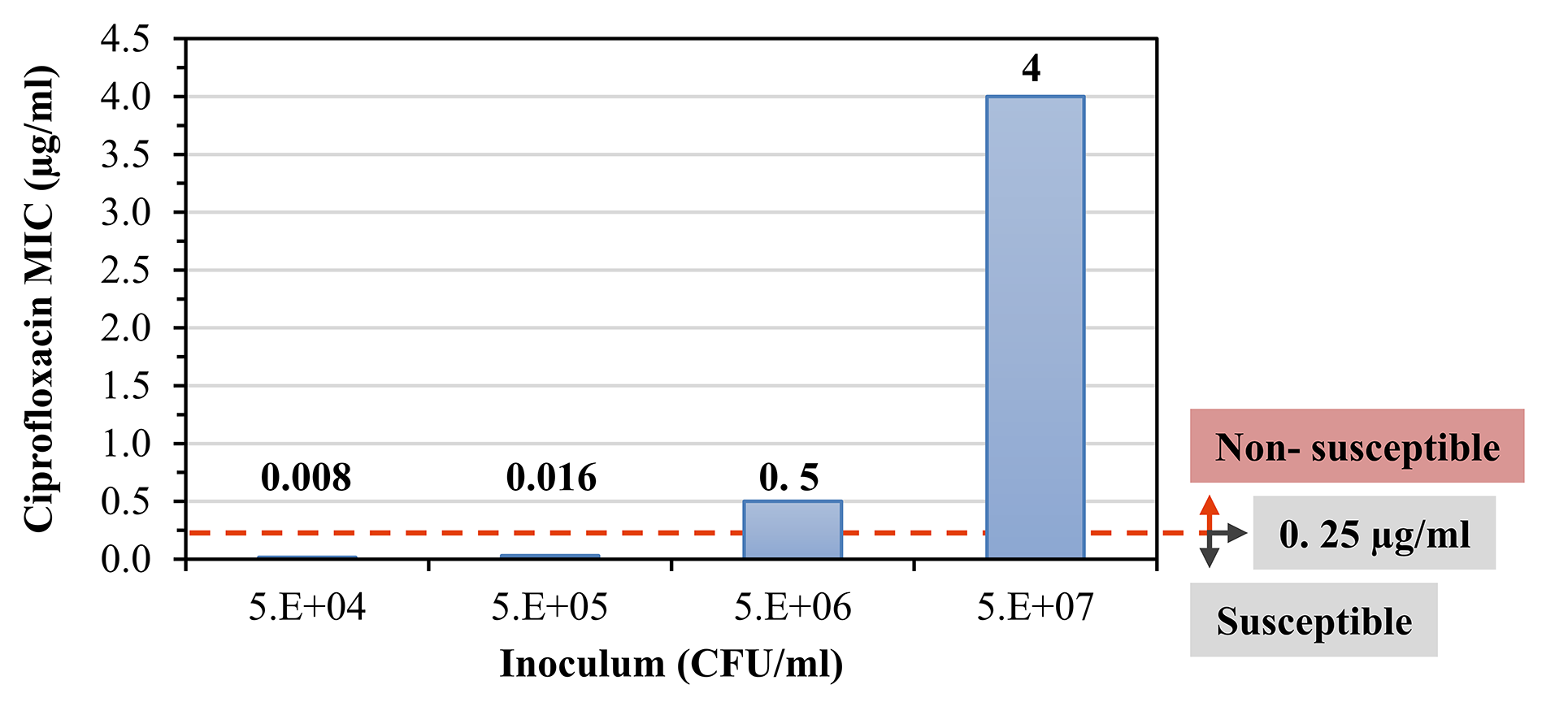

Supplement: Supplementary file 2 [file Image1.tif]

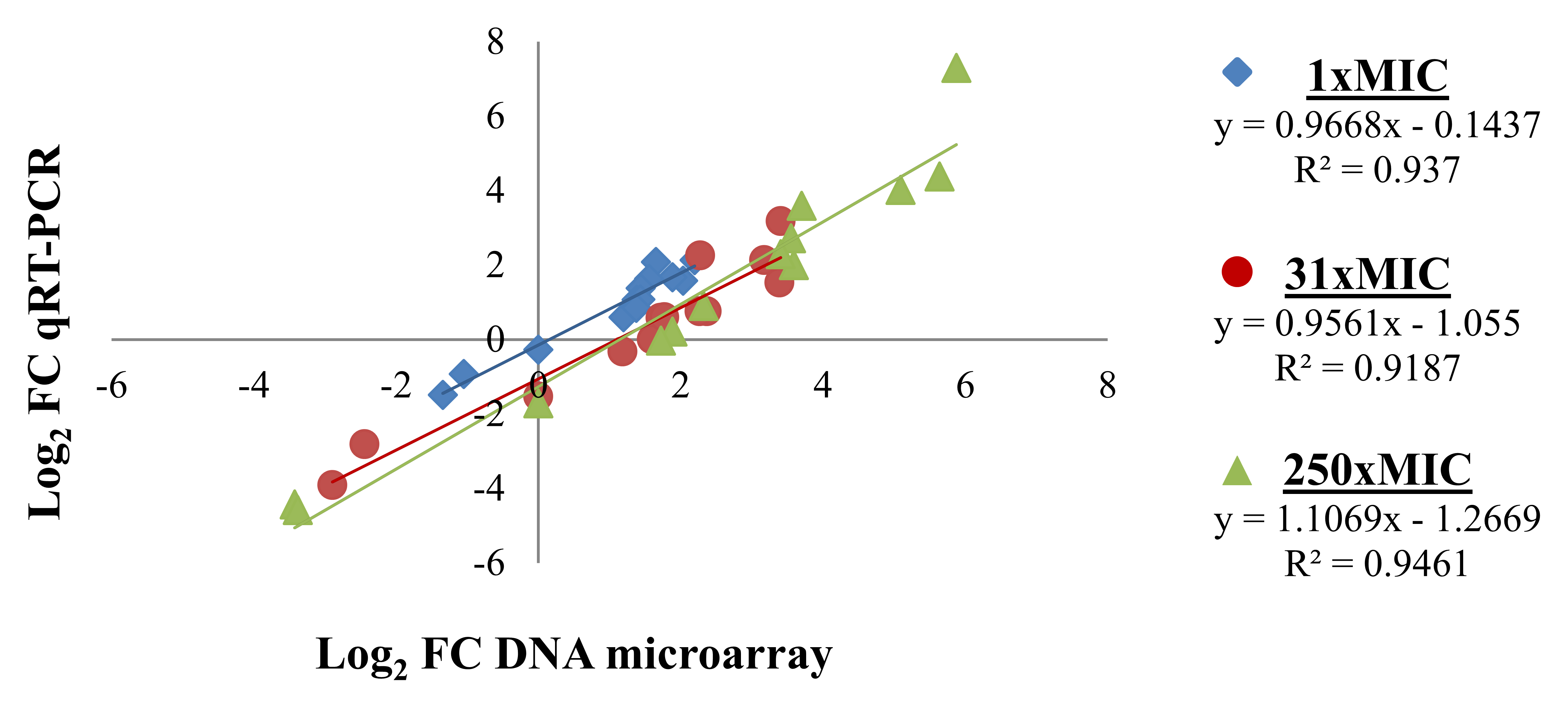

Supplement: Supplementary file 3 [file Image2.tif]

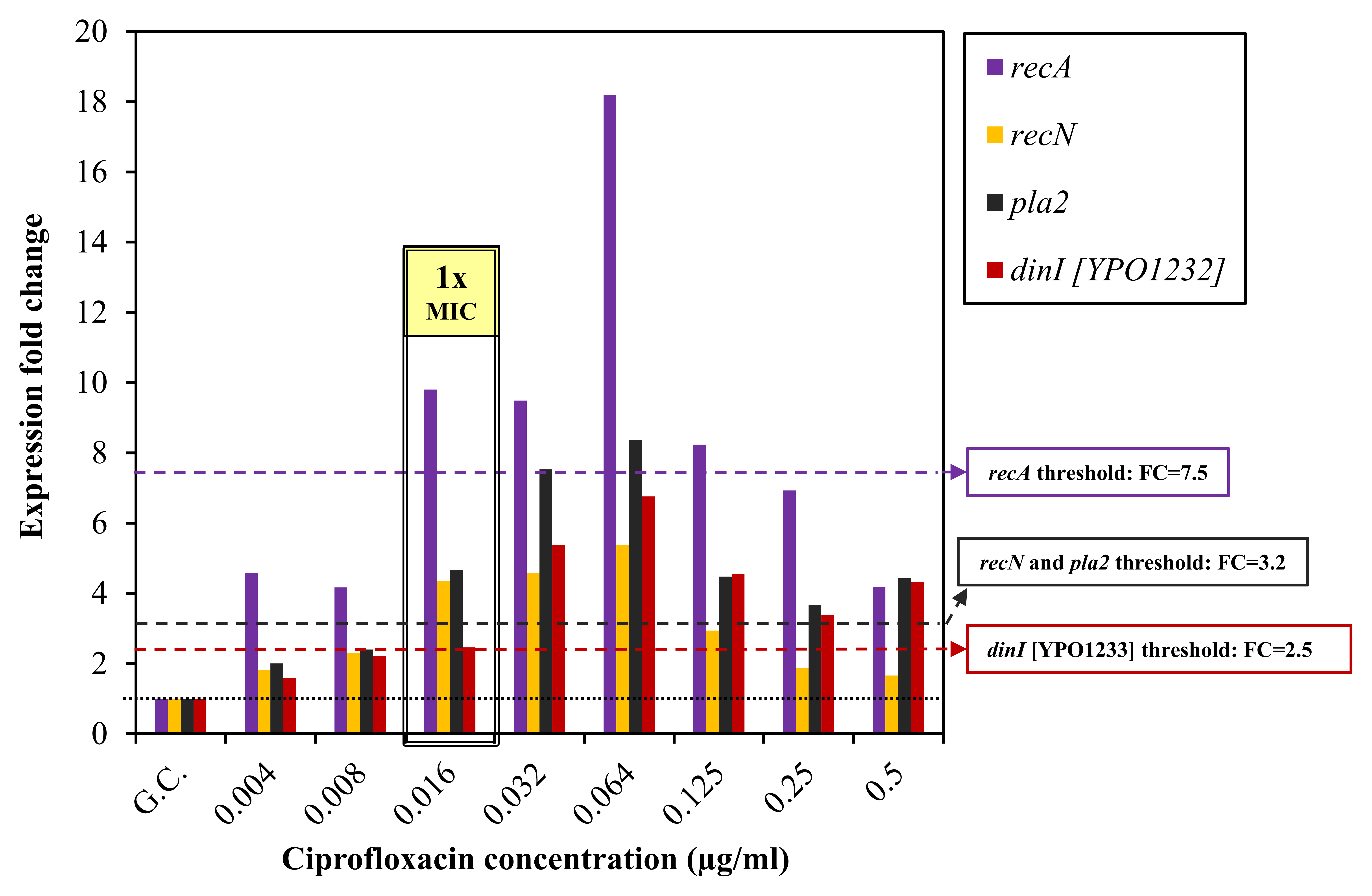

Supplement: Supplementary file 4 [file Image3.tif]

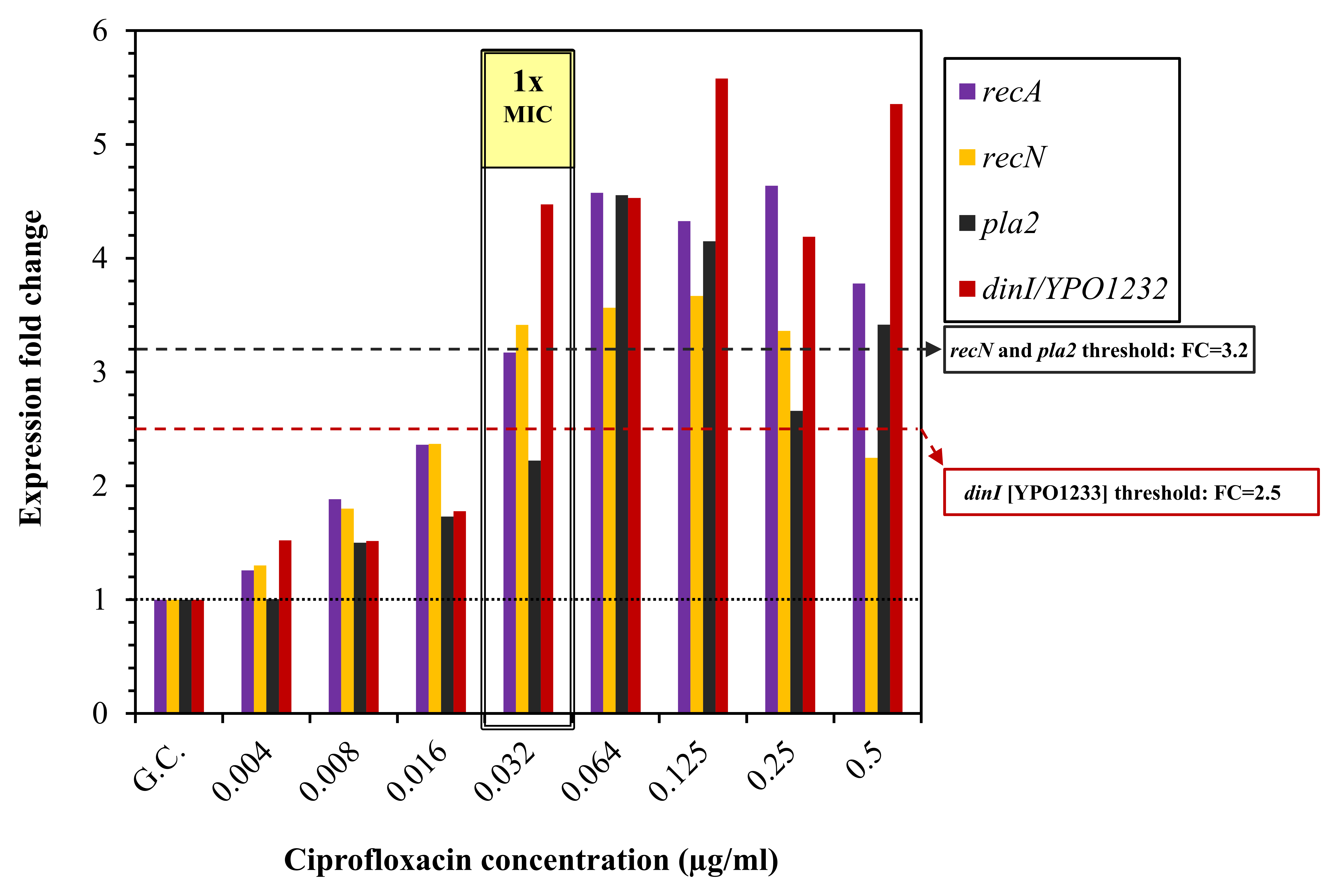

Supplement: Supplementary file 5 [file Image4.tif]
